# Willingness to participate in HIV vaccine efficacy trials in a population of fishing communities, Uganda

**DOI:** 10.1186/1742-4690-9-S2-P235

**Published:** 2012-09-13

**Authors:** A Nanvubya, J Mpendo, A Ssetaala, W Kidega, S Sigirenda, L Nielsen, N Kiwanuka

**Affiliations:** 1 UVRI-IAVI HIV Vaccine Program, Kampala, Uganda; 2 International HIV/AIDS Alliance in Uganda, Kampala, Uganda

## Background

With a number of preventive HIV vaccine candidates under pre-clinical and early-phase human studies, it is critical to intensify efforts to identify and prepare populations for potential efficacy trials. Willingness to participate (WTP) in HIV vaccine trials in most at risk populations for HIV infection is unknown especially in Sub-Saharan Africa. We assessed WTP in a potential HIV-1 vaccine efficacy trial population of fishing communities in Uganda.

## Methods

A community-representative random sample of 2,200 individuals, 18-49 years was selected at baseline from 8 fishing communities around Lake Victoria. After consent, data on HIV risk behaviors and WTP in future HIV vaccine trials were collected using a semi-structured questionnaire; venous blood was collected for HIV serology using rapid HIV tests as per the Uganda National algorithm.

## Results

Fifty percent of respondents were females and a majority (91%) had attained some level of education. Almost all respondents, 89% (1,953/2,192) expressed WTP in future HIV vaccine trials. Males were more willing to participate than females (p<0.01). WTP was associated with awareness of ongoing HIV vaccine research (p<0.01). Unwillingness to participate was attributed mainly to perceived fear of side effects (42%) and fear that the study vaccine may cause HIV/AIDS (26.3%). The overall HIV prevalence was 26.7%, lower among those who responded positively to WTP (26.8%) than those who did not (27.7%).

## Conclusion

WTP in HIV vaccine trials in this population is high but extensive education on HIV vaccine safety particularly on vaccine induced side effects and false positivity will be required in preparation for these trials. Because of the high HIV prevalence, this population should be targeted for future HIV vaccine trials.

